# Cancer-derived exosomal TRIM59 regulates macrophage NLRP3 inflammasome activation to promote lung cancer progression

**DOI:** 10.1186/s13046-020-01688-7

**Published:** 2020-08-31

**Authors:** Manman Liang, Xingwu Chen, Lijing Wang, Lilong Qin, Hanli Wang, Zhengui Sun, Wenying Zhao, Biao Geng

**Affiliations:** 1 Department of Infectious Diseases, Yijishan Hospital, The First Affiliated Hospital of Wannan Medical College, Wuhu, 241000 Anhui China; 2 Department of Respiratory Medicine, Yijishan Hospital, The First Affiliated Hospital of Wannan Medical College, 2 Zheshan West Road, Wuhu, 241000 Anhui China; 3 Department of Medical Oncology, Yijishan Hospital, The First Affiliated Hospital of Wannan Medical College, Wuhu, 241000 Anhui China

**Keywords:** Lung cancer, Exosomes, Macrophages, TRIM59, ABHD5, NLRP3

## Abstract

**Background:**

Exosomes are emerging as important mediators of the cross-talk between tumor cells and the microenvironment. The communication between tumor-derived exosomes and macrophages has a critical role in facilitating tumor progression. However, the mechanisms by which exosomes modulate tumor development in lung cancer are not fully understood.

**Methods:**

Short hairpin RNA mediated knockdown or exogenous expression of TRIM59 combined with in vitro and in vivo assays were performed to prove the functional significance of TRIM59. Western blotting, real-time PCR, co-immunoprecipitation, immunofluorescence (IF) staining assays, proximity ligation assay (PLA), ubiquitination assays, lactate secretion and lipid droplets content measurement, and rescue experiments were used to evaluate the mechanism. Lewis lung carcinoma (LLC) cells were injected via subcutaneously or tail vein into C57BL/6 wild-type (WT) and transgenic mice to assess the role of TRIM59 in vivo.

**Results:**

We demonstrated that tripartite motif-containing 59 (TRIM59) was expressed in lung cancer cells-derived exosomes, and can be transferred to macrophages through the exosomes. Activated macrophages by TRIM59 promote lung cancer progression in vitro and in vivo. Mechanistic investigations revealed that TRIM59 physically interacts with abhydrolase domain containing 5 (ABHD5) and directly induced the ubiquitination of ABHD5 and led to its proteasome-dependent degradation. ABHD5, an lipolytic co-activator, deficiency induced metabolic reprogramming and enabled NLRP3 inflammasome activation in macrophages. Further studies showed that the exacerbation of NLRP3 inflammasome activation by ABHD5 deficiency, provides a positive feedback loop to promote cancer progression by preferentially secrete the proinflammatory cytokine IL-1β.

**Conclusions:**

Collectively, these data indicate that tumor-derived exosomal TRIM59 converts macrophages to tumor-promoting functions of macrophages via regulating ABHD5 proteasomal degradation, to activate NLRP3 inflammasome signaling pathway to promote lung cancer progression by IL-1β secretion. Our findings also indicate that tumor-derived exosomal TRIM59 has an important role in intercellular communication for fostering an inflammatory microenvironment and promoting lung metastasis.

## Background

Lung cancer remains by far the most common cause of cancer mortality, which ranks among the most deadly cancers worldwide [1]. A growing number of evidences confirm that the tumor microenvironment contains diverse cell populations that interact with cancer cells and participate in all stages of tumorigenesis [2]. Tumor-infiltrating immune cells and immune responses within the tumor microenvironment are promising therapeutic targets [3]. Immune evasion represents a hallmark of cancer [4]. The majority of cancer immunotherapies, including immune checkpoint blockade therapy, aim to counteract immune evasion by shifting the balance in favor of immune activation, enabling T cell-mediated cancer cell elimination [4, 5]. However, only a subset of patients benefit from immunotherapies, emphasizing the need to identify the genomic and molecular determinants underpinning immune evasion [6]. Therefore, the discovery of new diagnostic biomarkers and a better understanding of molecular mechanisms underlying the tumor microenvironment of lung cancer are crucial.

During lung cancer, the tumor microenvironment consists of a heterogeneous collection of extracellular matrix, fibroblasts, perivascular cells, and, notably, is enriched in highly active immune cells [7]. Recent evidence suggests that exosomes are a vital communication medium between different cell types in the tumor microenvironment [8, 9]. Exosomes, as a novel mechanism of intercellular communication, can shuttle bioactive molecules from one cell to another, leading to the exchange of genetic information and reprogramming of recipient cells [10, 11]. Increasing evidence suggests that tumor cells release excessive amount of exosomes that promote tumor growth [10, 11]. In addition, tumor-derived exosomes signal immune cells in tumor microenvironment, helping tumor cells escape immune surveillance [12]. Accumulating evidence demonstrates that macrophages, the most abundant leukocyte population in lung cancer, have a critical role at each stage of cancer progression [13]. Such tumor-associated macrophages (TAMs) facilitate neoplastic transformation, tumor immune evasion and the subsequent metastatic cascade [13]. Thus, understanding the role of exosomes in the regulation of the TAMs response in cancer patients is important because TAMs are the major effector cells mediating anti-tumor immunity.

Tripartite motif (TRIM) family proteins, most of which have E3 ubiquitin ligase activities, have various functions in cellular processes including intracellular signaling, development, apoptosis, protein quality control, innate immunity, autophagy, and carcinogenesis [14]. The ubiquitin system is one of the systems for post-translational modifications, which play crucial roles not only as markers for degradation of target proteins by the proteasome but also as regulators of protein–protein interactions and of the activation of enzymes. Accumulating evidence has shown that TRIM family proteins have unique, important roles and that their dysregulation causes several diseases classified as cancer, immunological disease, or developmental disorders [14]. TRIM59 is a member of the TRIM protein superfamily, and has a TRIM or RBCC motif consisting of a RING-finger domain (R), a B-box domain (B), and a coiled-coil domain (CC). Increasing evidence showed that TRIM59 has been identified as contributing to tumor progression [15–18]. Our previous study has demonstrated that TRIM59 upregulated in lung cancer, is required for cancer cells survival and metastasis [19]. With the unraveling of the relationship between TRIM59 and tumors, TRIM59 is now being recognized as potential therapeutic targets for cancer. However, the mechanisms of TRIM59 promoting cancer metastasis and the communication between the tumor microenvironment and tumor cells are still waiting for exposure.

In this study, our findings unveil a new sight between tumor cells and macrophages: TRIM59 are directly shuttled from cancer cells via exosomes to macrophages, promoting ABHD5 proteasomal degradation and inducing NLRP3 (NLR family protein containing a pyrin domain 3) inflammasome activation and promoting IL-1β secretion, in turn promoting cancer cells proliferation and invasion. Thus, the data implicate TRIM59 as a target for exosome-mediated tumor immune evasion.

## Materials and methods

### Cell culture

The human cell lines H1299, A549, THP-1, lewis lung carcinoma, and HEK293 were purchased from the Chinese Academy of Science Cell Bank (Shanghai, China). They have been authenticated by a STR DNA profiling analysis and routinely examined for mycoplasma contamination. The cell lines were maintained at 37 °C in a humidified atmosphere of 5% CO2 with DMEM medium containing 10% fetal bovine serum (FBS) with 100 U/ml penicillin G and 100 μg/ml streptomycin sulfate. Bone marrow derived macrophages (BMDMs) were obtained as previously described [20]. To induce differentiation into macrophages, THP-1 cells (1 × 10^6^) were incubated with 100 ng/ml phorbol 12-myristate 13-acetate (PMA) (Sigma-Aldrich) for 24–48 h.

### Exosome isolation and identification

For exosomes isolation, we first transplanted equal number of different cells into 10 cm plates and changed the culture medium with fresh DMEM-supplemented serum, which was depleted of exosomes by centrifugated at 12,000×g overnight. After 48 h, conditioned medium (CM) was collected and filtrated through 0.22 μm filters (Millipore). Exosomes in CM were isolated by a precipitation method using Total Exosome Isolation Reagent (Invitrogen, Cat: #4478359) according to the manufacturer’s instruction. Briefly, Transfer the required volume of cell-free culture media to a new tube and add 0.5 volumes of the Total Exosome Isolation (from cell culture media) reagent. Mix the culture media/reagent mixture well by vortexing. Incubate samples at 2 °C to 8 °C overnight. After incubation, centrifuge the samples at 10,000×g for 1 h at 2 °C to 8 °C. Aspirate and discard the supernatant. Exosomes are contained in the pellet at the bottom of the tube. Resuspend the pellet in a convenient volume of 1X PBS.

Isolated exosomes were mixed with 4% paraformaldehyde. Exosomes were then dropped onto formvar carbon-coated electron microscopy grids and fixed with 1% glutaraldehyde for 10 min. Samples were negatively stained with 2% uranyl acetate solution. Images were obtained using a transmission electron microscopy.

### Exosome labeling and tracing

Purified exosomes isolated from the culture medium were collected and labeled with PKH67 Green Fluorescent membrane linker dye (Sigma-Aldrich) according to manufacturer’s instructions. Then, the labeled exosome pellets were resuspended and added to the unstained macrophages for exosomes uptake studies. After incubation for 0 h, 4 h, 8 h or 12 h at 37 °C, cells were observed by fluorescence microscopy.

### Western blotting

Exosomes or cells were lysed with RIPA buffer containing a complete protease inhibitor tablet (Roche). Proteins were separated by SDS-PAGE gel and transferred onto polyvinylidene difluoride membranes. After blocked in 5% skim milk for 30 min, membranes were probed with various primary antibodies overnight at 4 °C, followed by incubation with horseradish peroxidase–linked secondary antibodies for 1 h at room temperature, and visualized with electrochemiluminescence by the chemiluminescence instrument. The following antibodies were used: TRIM59 (Invitrogen, Cat: PA5–38726), ABHD5 (Invitrogen, Cat: PA5–78704), CD63 (Invitrogen, Cat: 10628D), CD81 (Invitrogen, Cat: 16–0811-82), TSG101 (Invitrogen, Cat: PA5–82236), SLC16A4(MCT4) (Invitrogen, Cat: PA5–80008), β-actin (Cell Signaling Technology, Cat: #3700), HA-Tag (Cell Signaling Technology, Cat: #3724), Myc-Tag (Cell Signaling Technology, Cat: #2276), IL-1β (Cell Signaling Technology, Cat:#12703), Cleaved-IL-1β (Cell Signaling Technology, Cat: #83186), Caspase-1(Cell Signaling Technology, Cat: #3866), and Cleaved Caspase-1 (Cell Signaling Technology, Cat: #89332).

### RNA extraction and qRT-PCR

Total RNA was extracted from cells using Trizol reagent (Invitrogen) according to the manufacturer’s instructions. Aliquots of 1 μg of total RNA were reverse transcribed using SuperScript II Reverse Transcriptase (Invitrogen) and oligo-dT (18)-primers (Invitrogen). The real time PCR reaction was performed using SYBR Green Master Mix kit in ABI Prism 7000 Sequence Detection System (Applied Biosystems) according to the manufacturer’s instructions. Glyceraldehyde-3-phosphate dehydrogenase (GAPDH) was amplified as an internal standard. The Taqman probe for IL-1β (Cat: HP100210) and GAPDH (Cat: HP100003) were purchased from Sino Biological Inc. (Beijing, China). The primers for mouse TRIM59: 5′-ATGCACAATTTTGAGGAGGAG-3′ (forward) and 5′-TCAACGAGAAACTATTTTCC-3′ (reverse). The expression levels of the mRNAs were reported as fold changes vs. control.

### Immunofluorescence (IF) staining

THP-1 macrophages, TAMs, and BMDMs used in the tests was seeded on cover slides in 24-well plates and incubated overnight. Cells were fixed in 4% paraformaldehyde for 30 min, permeabilized with 1% Triton X-100 for 20 min, blocked in 5% bovine serum albumin for 60 min, and incubated with primary antibodies against TRIM59 and ABHD5 overnight at 4 °C, followed by an Alexa Fluor 594-conjugated secondary antibody and Alexa Fluor 488-conjugated secondary antibody for 30 min at RT. Nuclei were counterstained with 4′,6-diamidino-2-phenylindole dihydrochloride (DAPI, Sigma-Aldrich) at room temperature for 5 min. IF signals were captured using a laser confocal microscopy.

### Plasmid constructs and RNA interference

HA-TRIM59 (Cat: HG25849-NY), TRIM59 (Cat:HG25849-UT), Myc-ABHD5 (Cat: HG14216-CM), and ABHD5 (Cat: HG14216-UT) expression plasmid were purchased from Sino Biological Inc. Stable TRIM59 was up-regulated using Human TRIM59 lentiviral particles with C terminal GFP Spark tag (Sino Biological Inc.; Cat: HG25849-ACGLN) as the manufacturer’s instructions.

Scrambled, human ABHD5 short hairpin RNA (shRNA) were obtained from Shanghai Genechem Co., Ltd. (Shanghai, China) and the following target sequences: CCGGGCAGCGTTTAAGGCCTGATTTCTCGAGAAATCAGGCCTTAAACGCTGCTTTTTG. TRIM59 shRNA was described previously [19]. To elucidate the role of TRIM59 as an E3 ligase, we generated an E3 ligase-defective TRIM59 by using the KOD-Plus-Mutagenesis kit (Toyobo, cat: SMK-101) and verified by performing DNA sequencing. Plasmids were transiently transfected into HEK293 cells or THP-1 cells with lipofectamine 3000 reagents according to the manufacturer’s instructions.

### Proximity ligation assay (PLA)

PLA was performed using the Duolink In Situ Red Kit purchased from Sigma-Aldrich (DUO92101) in accordance with the manufacturer’s instruction. Briefly, transfected cells were washed once with ice cold PBS, followed by fixation with 4% paraformaldehyde for 15 min at room temperature. Fixed cells were then washed three times with PBS and permeabilized with 0.5% Triton X-100 containing PBS for 10 min. Permeabilized cells were blocked with 5% normal goat serum for 1 h at room temperature. The cells were then incubated with primary antibodies diluted in 10% normal goat serum supplemented with 0.1% Tween at 4 °C overnight. Following the incubation, the cells were washed three times with PBS and then incubated with two PLA probes (Duolink In Situ PLA Probes Anti-rabbit PLUS and Anti-Mouse MINUS, Sigma-Aldrich) for 1 h at 37 °C. After probe incubation, the samples were incubated in ligation solution for 1 h at 37 °C. After ligation, cells were washed with Wash Buffer A and incubated in the amplification solution for 2 h at 37 °C. Cells were then serially washed twice in 1 × Wash Buffer B, 0.01 × Wash Buffer B once, and PBS once, followed by incubation with secondary antibodies for 1 h at room temperature. Finally, cells were washed three times with PBS and mounted in Duolink In Situ Mounting Medium supplemented with DAPI. Fluorescence images were obtained with a confocal microscope.

### Co-immunoprecipitation(Co-IP)

Co-IP assays were performed according to standard protocols. For the Co-IP of Myc-ABHD5 and HA-TRIM59 proteins, anti-Myc and anti-HA agarose beads (20 μl) were used to pull down Myc-ABHD5 and HA-TRIM59, respectively. The protein-antibody complexes recovered on beads were subjected to western blotting using appropriate antibodies after separation by SDS-PAGE. The purified IgG was used as a negative control.

### Ubiquitination assay

For ubiquitination assays, cells were transfected with vectors, including vectors expressing Myc-ABHD5, HA-TRIM59 and His-Ub, respectively, for 24 h. Cells were then treated with MG132 (10 μM) (Sigma-Aldrich) for 8 h, and the levels of Myc-ABHD5 ubiquitination was determined by IP with an anti-Myc antibody followed by western blot assays with an anti-His antibody (Cell Signaling Technology, Cat: #3936).

### Protein half-life analysis

A cycloheximide (CHX) (Sigma-Aldrich) blocking analysis was performed to determine the half-life of ABHD5. Cells were incubated with CHX (50 μg/ml) for various times, and ABHD5 was detected by western blot analysis. The density of the immunoreactive bands corresponding to ABHD5 and β-actin were measured. The level of ABHD5 was quantified by normalization with β-actin, and the percentage of remaining ABHD5 was plotted.

### Cell proliferation assay

LC cells proliferation was assessed using the EdU Assay Kit (RiboBio Inc., China) according to the manufacturer’s instructions.

### Transwell invasion assays

The effect of conditioned medium of macrophages on the invasion of cancer cells was determined by using transwell 24-well plates (8 μm pore size, BD Biosciences). Cells were allowed to invade through the Matrigel (BD Biosciences) for 48 h. Migrated cells were fixed with 4% paraformaldehyde and stained with 1% crystal violet. For each chamber, three fields were randomly chosen and cells were counted.

### Lactate secretion and lipid droplets content measurement

THP-1 macrophages at different treatments and regular culture medium was discarded and replaced with 2 ml fresh culture medium then culture for another 24 h, the lactate concentration was measured by lactate assay kit (Sigma-Aldrich, Cat: MAK064).

THP-1 macrophages (5 × 10^5^ cells per well) at different treatments were rinsed in PBS once (1 min) and then fixed in 4% paraformaldehyde for 10 min. After rinsing with PBS for 3 times, 3 min each, cells were stained with Lipid (Oil Red O) Staining Kit (Sigma-Aldrich, Cat: MAK194). For lipid droplets content quantification, Oil Red O dye was extracted with DMSO and optical density (OD) was detected using a spectrophotometer at 496 nm.

### Measurement of cytokine production

THP-1 macrophages or BMDMs were seeded in 24-well plates and cultured overnight. After priming with 1 μg/ml LPS for THP-1 macrophages or 100 ng/ml LPS for mouse BMDMs and stimulating with Nigericin (50 μM), ATP (5 mM), Alum (200 μg/ml), poly(dA:dT) (200 ng/ml) or flagellin (200 ng/ml), the supernatants were collected, and the concentrations of IL-1β were measured using ELISA kits (R&D Systems, Cat: #201-LB), according to the manufacturer’s instruction.

### Multiplex immunohistochemistry

A LC tissue microarray containing 90 cases of LC and paired adjacent non-cancerous tissue was purchased from Shanghai Outdo Biotech (HLugAde180Sur01). For mIHC staining, Opal 4-color fluorescent IHC kit (Akoya Biosciences, Cat: #NEL810001KT) was used. First, the concentration and the application order of the three antibodies were optimized, and the spectral library was built based on the single-stained slides. The slides were first deparaffinized by xylene and ethanol and antigen retrieval was performed by microwave. After incubating with 3% H2O2 (freshly made) for 10 mins, the tissues were blocked in blocking buffer for another 10 mins at room temperature. Then the tissues were incubated by primary antibody, secondary-HRP (Cell Signaling Technology) and Opal working solution (Akoya Biosciences). The slides then were mounted with ProLong Gold Antifade Reagent with DAPI, and were scanned using confocal microscopy. Then several 20x fields (3–5 per sample) were analyzed at regions of interest (10–15 per field) identified along tumor and stromal zones. TRIM59 and IL-1β mean fluorescence intensities (MFIs) were measured, respectively. The results were confirmed by two experienced pathologists blinded to the clinicopathological parameters. Image J software was used for image quantification.

### Isolation of peritoneal macrophages (PMs)

Each mouse was injected (i.p.) with 2 ml of 3% thioglycolate on day 1 and killed on day 3. After i.p. injection of 5 ml DMEM cell culture medium containing 10% FBS, as well as penicillin and streptomycin, the peritoneal cells were collected in cell culture dishes. Two hours later, the floating cells were removed by washing the cells with phosphate-buffered saline (PBS). The attached cells were considered to be PMs (purity: 90%) and were subjected to further experiments.

### Isolation TAMs by magnetic-activated cell sorting (MACS) separation

Fresh lung tumor tissues were prepared as single cell suspensions, by minced in the DMEM with 0.1% collagenase I (1 h, 37 °C) and filtered using a 40 μm cell strainer (BD Falcon). Cells in suspensions were stained for 30 min at 4 °C with anti-mouse FITC-F4/80 antibody (eBiosience). After rigorous washing with 0.5% BSA and 2 mM EDTA in PBS, cells were incubated with an anti-FITC Microbeads (Miltenyi Biotec) for 20 min at 4 °C. F4/80+ cells were sorted by MACS separation according to the manufacturer’s instruction (Miltenyi Biotec), then F4/80+ cells (TAMs) and pass-through cells (mainly tumor cells) were prepared.

### Animal experiments

The transgenic mice with macrophages overexpression of TRIM59 (Tg^TRIM59^) were constructed (The cDNA of mouse TRIM59 was subcloned into a construct containing the human CD11b promoter to drive macrophage-specific gene expression, on a C57BL/6 J background, Model Animal Research Center, Nanjing University). We utilized 6 to 8-week-old C57BL/6 J WT and Tg^TRIM59^ mice in this study. The mice were subcutaneously injected with lewis lung carcinoma (LLC) cells (5 × 10^6^ cells per mouse). The tumor size of mice was measured. After 16 days, the mice were sacrificed and their tumors tissues were determined for histological examination. Tumor volume was determined using the formula: Volume = width × length × (width + length)/2.

In vivo metastasis, the C57BL/6 WT and transgenic mice were intravenously injected with LLC cells (5.0 × 10^6^ cells per mouse) via the tail vein. The mice were sacrificed 14 days after tumor inoculation. All the suspicious lung metastasis sites were evaluated by histologic examination. All animal experiments were approved by the Institutional Animal Care and Use Committee and performed according to the institution’s guidelines and animal research principles.

### Statistical analysis

All values are expressed as the mean ± SEM. Statistical analysis were performed with GraphPad Prism 7. Student’s *t* tests were used to evaluate continuous variables between subgroups. Values of *P* < 0.05 were considered to be statistically significant.

## Result

### TRIM59 is highly expressed in exosomes derived from lung cancer cells and can be transferred to macrophages through the exosomes

**Fig. 1:**
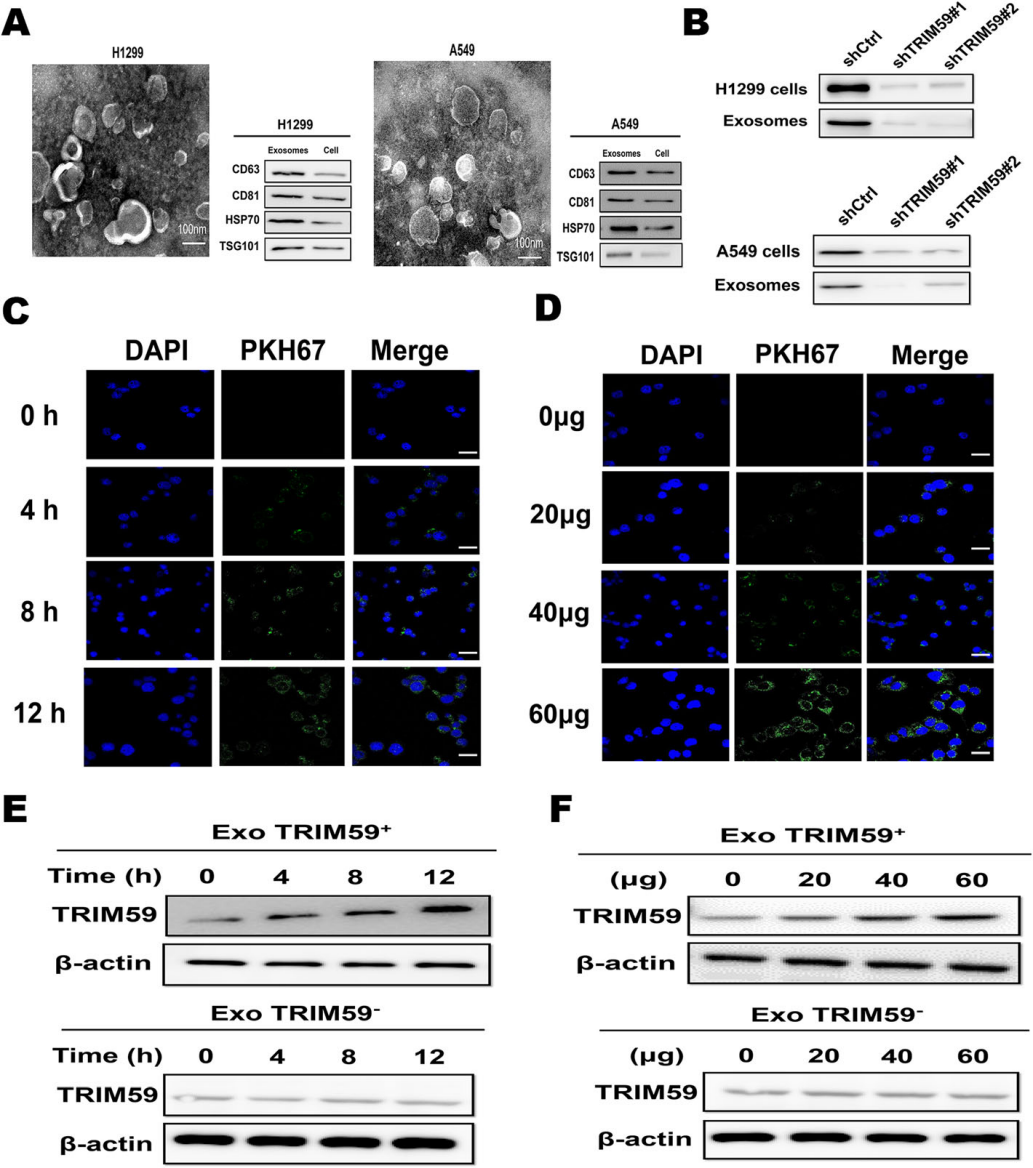
Exosomal TRIM59 is characteristically secreted by lung cancer cells and transferred to and internalized by macrophages via exosomes. **a**. Exosomes released by H1299 and A549 cells were detected by electron microscopy. Scale bar, 100 nm. Immunoblotting assay of indicated proteins in exosomes from H1299 and A549. **b**. Western blot evaluations were used to evaluateTRIM59 expression in both LC cells (H1299 and A549) exosomes and LC cells treated with TRIM59 shRNA or scrambled controls. **c**-**d**. Representative immunofluorescence image shows the internalization of PKH67-labeled H1299-derived exosomes (green) by macrophages. Confocal imaging showed the delivery of PKH67-labeled exosomes (green) to macrophages. Green dots represented delivered exosomes. Scale bar, 150 μm. **e**-**f**. THP-1 macrophages cells were incubated with exosomes for the noted periods of time or the noted doses. Western blot evaluations were used to evaluate TRIM59

Exosomes derived from H1299 and A549 cells were purified using a total exosome isolation reagent and identified by transmission electron microscopy (TEM). TEM revealed cup-shaped structures, and showed a mean particle size of 50–100 nm diameter structures that are typical of exosomes (Fig. 1a). Moreover, exosomes markers CD63, CD81, HSP70, and TSG101 proteins were positively expressed in these vesicles (Fig. 1a). Recently, studies indicated that exosomes contain a variety of biologically active molecules including proteins, and that exosomal proteins profiles resemble those of the parent cells [9, 11]. We previously found that TRIM59 is closely correlated with oncogenesis and metastasis of lung cancers [19]. To elucidate whether TRIM59 was expressed in exosomes derived from lung cancer cells (H1299 and A549), we detected the expression of TRIM59 in the exosomes derived from A549 and H1299 cell lines, in which TRIM59 expression levels significantly elevated [19]. As is shown in Fig. 1b, TRIM59 was detected in the exosomes of H1299 and A549 cells, whereas the levels of TRIM59 in both LC cells and exosomes were strongly decreased by transfection of TRIM59 short hairpin RNA (shRNA).

Once secreted, exosomes deliver biological information to neighbouring or distant cells, thus modulating communication between tumor cells and the surrounding microenvironment. Macrophages are the most abundant infiltrative immune-related stromal cells present in and around tumors [13]. It was shown that proteins can be transferred from cells to cells via exosomes [21]. Thus, we detected whether TRIM59 could be transferred from lung cancer cells to macrophages via exosomes. To examine the transfer of exosomal TRIM59 to recipient cells, THP-1 macrophages were incubated with PKH67-labelled exosomes derived from H1299 and A549 cells. These exosomes were found to enter macrophages in a time-dependent and concentration-dependent manner (Fig. 1c, d, Figure S1A, B). Moreover, green fluorescent protein (GFP)-tagged TRIM59 was expressed in H1299 and A549 cells and the exosomes derived from H1299 and A549 cells were isolated and incubated with mixed THP-1 macrophages. As was expected, the GFP-tagged TRIM59 was detected in THP-1 macrophages (Figure S1C, D). To further confirm exosomal TRIM59 internalization by macrophages, we also evaluated TRIM59 protein levels in macrophages. Results showed that macrophages incubated with exosomes derived from H1299 and A549 cells (ExoTRIM59^+^) had higher TRIM59 level than that with exosomes derived from TRIM59-knockout H1299 and A549 cells (ExoTRIM59^−^) in a time-dependent and concentration-dependent manner (Fig. 1e, f, Figure S1E, F). Taken together, these results clearly demonstrated that TRIM59 is expressed in lung cancer cells-derived exosomes, and can be transferred to macrophages through the exosomes.

### TRIM59 interacts with ABHD5

**Fig. 2:**
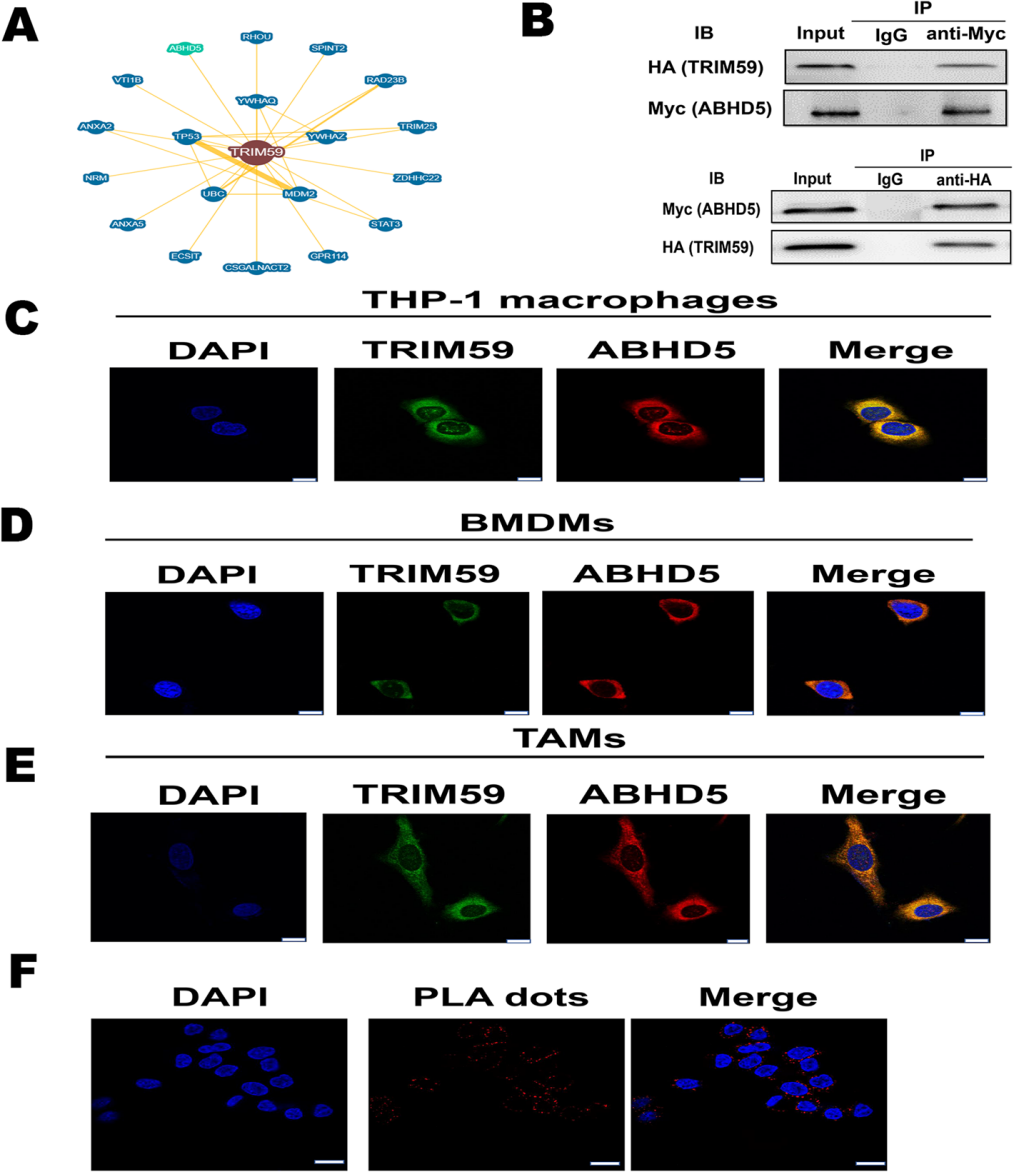
TRIM59 binds and co-localizes with ABHD5. **a**. Network graph representation of interaction from the BioGRID for TRIM59. Users can select the ‘Network’ tab from the ‘Switch View’ menu to view interactions data when available. **b**. HEK-293 cells were transfected with HA-tagged TRIM59 and Myc-tagged ABHD5, lysates were prepared and immunoprecipitated (IP) with either anti-HA or anti-Myc, and the precipitates were evaluated using immunoblot (IB) analysis as noted. **c**-**e**. Immunohistochemical demonstration of the co-localization of TRIM59 and ABHD5 in THP-1 macrophages, BMDMs, and TAMs. Fluorescence images were counterstained with 4′6-diamidino-2-phenylindole (DAPI) for nucleus identification. Scale bars, 10 μm. **f**. THP-1 macrophages cells were transiently transfected with HA-tagged TRIM59 and Myc-tagged ABHD5. Rabbit anti-HA and Mouse anti-Myc antibody were used for the proximity ligation assay. Red dots present the interaction of TRIM59 with ABHD5. Scale bars, 20 μm

Recent studies have indicated that TRIM proteins changes in expression levels could disrupt the balance of TRIM proteins in the cell and alter ubiquitination of various proteins to produce irregular cellular signaling that could lead to tumorigenesis [14]. An accumulating body of evidence has demonstrated that some TRIM proteins function as E3 ubiquitin ligases in specific ubiquitin-mediated protein degradation pathways. These functional characteristics led us to further understand which protein TRIM59 binds to and how this interaction affects the physiological function of the target protein. Affinity capture mass spectrometry showed that TRIM59 could interact with abhydrolase domain containing 5 (ABHD5) protein, an activator of triglyceride hydrolysis [22] (Fig. 2a). Previous studies demonstrated that tumor-associated macrophages exhibit heterogeneous expression of ABHD5, with migratory TAMs expressing lower levels of ABHD5 compared to the non-migratory TAMs [23]. These data led us to investigate whether TRIM59 as a RING domain-containing E3 ligase plays a physiological role in regulating the ubiquitination and degradation of ABHD5. Further documentation of the interaction between TRIM59 and ABHD5 was obtained with co-immunoprecipitation (Co-IP), colocalization, and proximity ligation assay (PLA) assays. In the former, we co-expressed HA-tagged TRIM59 and Myc-tagged ABHD5 in human 293 cells. HEK-293 cells were transfected with both of these moieties and subjected to immunoprecipitation (IP) with antibodies to one moiety, and the precipitate was then analyzed via western blotting using antibodies to the other moiety. In these experiments, the two moieties always traveled together with IP using antibodies against HA (TRIM59) always precipitating Myc (ABHD5) and vice versa (Fig. 2b). To further confirm the interaction between TRIM59 and ABHD5, we carried out confocal microscopy analysis to show that TRIM59 predominantly co-localized with ABHD5 in THP-1 macrophages, mouse bone marrow derived macrophages (BMDMs), and TAMs (Fig. 2c-e). To investigate direct association of TRIM59 and ABHD5, we performed proximity ligation assays. PLA analysis demonstrated TRIM59 directly associated with ABHD5 in THP-1 macrophages (Fig. 2f). When viewed in combination, these studies demonstrated that TRIM59 physically interacts with ABHD5.

### TRIM59 promotes ubiquitination and degradation of ABHD5

**Fig. 3:**
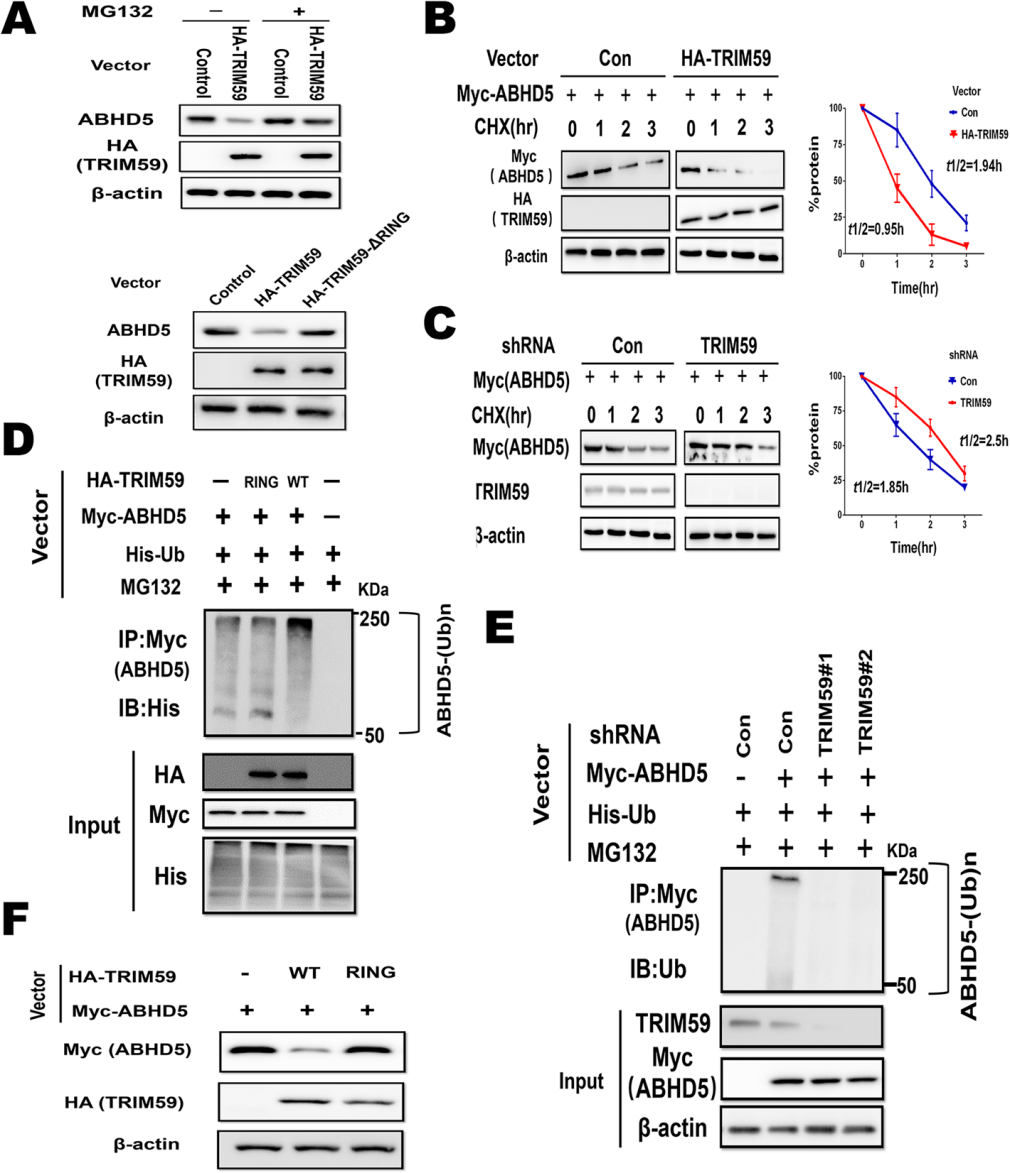
TRIM59 promotes ABHD5 protein degradation through ubiquitination. **a**. Proteasome inhibitor MG132 inhibited the downregulation of ABHD5 protein levels induced by HA-TRIM59 expression in HEK-293 cells. HEK-293 cells were transfected with HA-TRIM59 or HA-TRIM59-∆RING vector. The indicated proteins were measured by western blotting. **b**. HA-TRIM59 expression decreased ABHD5 protein half-life in cells. HEK-293 cells with ectopic HA-TRIM59 expression and control cells were transfected with the Myc-ABHD5 vector. The cells were treated with 50 μg/ml CHX for indicated time periods before being collected for western blot assays. **c**. Knockdown of endogenous TRIM59 increased Myc-ABHD5 protein half-life in THP-1 macrophages. **d**. The effects of expression of HA-TRIM59 and its mutants on ubiquitination of Myc-ABHD5 in HEK-293 cells analyzed by in vivo ubiquitination assays. **e**. Knockdown of endogenous TRIM59 decreased the ubiquitination of Myc-ABHD5 in THP-1 macrophages analyzed by in vivo ubiquitination assays. **f**. Mutations of TRIM59 that impaired TRIM59’s ubiquitination activity impaired the ability of HA-TRIM59 to degrade Myc-ABHD5 protein in THP-1 macrophages

To investigate whether TRIM59 negatively regulates ABHD5 through ubiquitin-proteasome degradation, HEK-293 cells were transfected with the HA-TRIM59 vector were treated with the proteasome inhibitor MG132, and ABHD5 protein levels were analyzed. MG132 treatment largely abolished the inhibitory effect of TRIM59 on ABHD5 protein levels in cells (Fig. 3a). It has been reported that the E3 ubiquitin ligases of TRIM59 is required for ubiquitin ligase activity of TRIM59 [14]. To elucidate the role of TRIM59 as an E3 ligase, we generated an E3 ligase-defective TRIM59 mutant (TRIM59-∆RING), in which the N-terminal RING domain was deleted and the potential E3 ubiquitin ligase activity was deprived. As shown in Fig. 3a, an increase in HA-TRIM59 expression resulted in decreased ABHD5 protein expression, which was not observed for HA-TRIM59-∆RING, indicating that the E3 ligase activity of TRIM59 is involved in ABHD5 degradation. Together, these results strongly suggest that proteasomal degradation of ABHD5 is mediated by TRIM59 which regulates ABHD5 protein levels in a manner that is dependent on its E3 ligase activity.

To investigate whether TRIM59 affects protein stability of ABHD5, the protein half-life of ABHD5 was analyzed. HEK-293 cells with HA-TRIM59 expression and their control cells were transduced with the Myc-ABHD5 expression vector before they were treated with protein synthesis inhibitor cyclohexamide (CHX) for different time periods. Compared with control cells transduced with the empty vector, cells transduced with the HA-TRIM59 vector exhibited reduced half-life of Myc-ABHD5 protein (Fig. 3b). Importantly, knockdown of endogenous TRIM59 increased the half-life of Myc-ABHD5 protein in THP-1 macrophages (Fig. 3c).

To investigate whether TRIM59 promotes ABHD5 degradation through ubiquitination, in vivo ubiquitination assays were employed. HEK-293 cells were co-transfected with HA-TRIM59 or mutant HA-TRIM59-∆RING vectors together with vectors expressing Myc-ABHD5 and His-ubiquitin (His-Ub), respectively. Cells with HA-TRIM59 expression displayed increased ubiquitination of Myc-ABHD5 compared with cells transfected with the control vector (Fig. 3d). Notably, its inactive mutant markedly reduced the ability of TRIM59 to ubiquitinate Myc-ABHD5 in HEK-293 cells (Fig. 3d). Furthermore, knockdown of endogenous TRIM59 decreased Myc-ABHD5 ubiquitination in THP-1 macrophages (Fig. 3e). These results suggested that TRIM59 mediates ABHD5 ubiquitination, leading to its degradation. We further examined whether above-mentioned TRIM59-∆RING mutants can impair TRIM59’s ability to degrade ABHD5 in cells. As shown in Fig. 3f, TRIM59-∆RING mutants largely abolished the ability of TRIM59 to degrade Myc-ABHD5 in THP-1 macrophages. Taken together, these results indicated that the E3 ubiquitin ligase TRIM59 downregulates ABHD5 through ubiquitination and proteasomal degradation.

### ABHD5 deficiency promotes NLRP3 inflammasome activation in macrophages

**Fig. 4:**
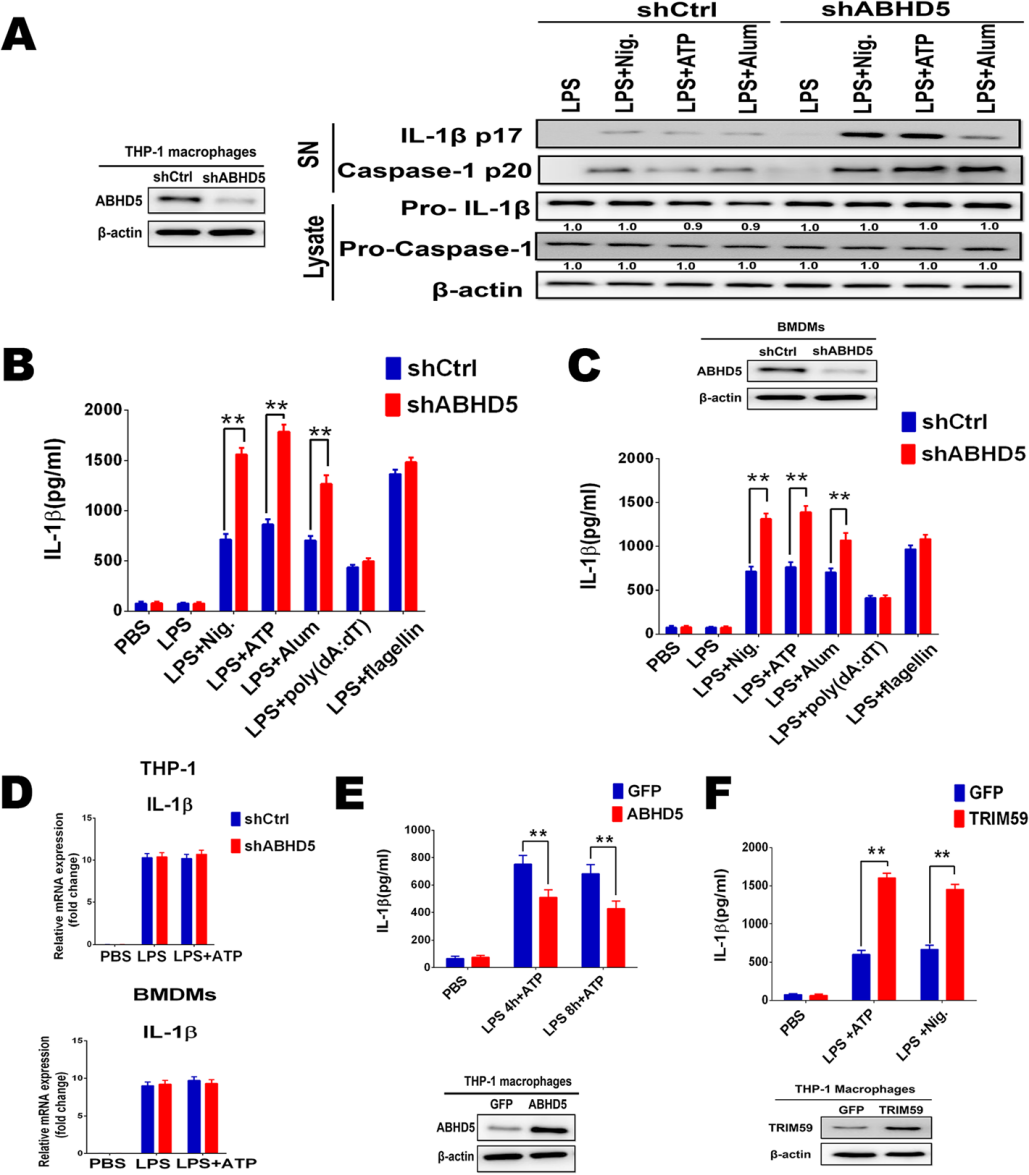
ABHD5 deficiency promotes NLRP3 inflammasome activation in macrophages. **a**. Western blot analysis of ABHD5 expression in macrophages transfected with scrambled control shRNA or ABHD5 shRNA for 36 h. Immunoblot of the IL-1β, the pro-caspase-1 and cleaved caspase-1 in the supernatants (SNs) or cell lysates of ABHD5-silenced THP-1 macrophages, primed with LPS, and then stimulated with Nigericin (Nig.), ATP or Alum. β-actin served as a loading control. Quantification of Western blotting were performed with the Image J software. Numbers below each blot indicate relative band intensity normalized to β-actin. **b**. ELISA of IL-1β in supernatants from THP-1 macrophages silenced of ABHD5, primed with LPS for 8 h, and followed by stimulation with ATP, Nig., Alum, poly(dA:dT) or flagellin for 30 min. **c**. *ELISA* of IL-1β in supernatants from BMDMs silenced of ABHD5, primed with LPS for 8 h, and followed by stimulation with ATP, Nig., Alum, poly(dA:dT) or flagellin for 30 min. **d**. RT-PCR analysis of IL-1β mRNA expression in macrophages transfected with shRNA as indicated and stimulated as indicated. **e.** ELISA of IL-1β in supernatants from THP-1 cells infected with lentiviral vectors expressing ABHD5 or GFP control, primed with LPS for various times, and followed by stimulation with ATP for 30 min. **f**. ELISA of IL-1β in supernatants from THP-1 cells infected with lentiviral vectors expressing TRIM59 or GFP control, primed with LPS, and followed by stimulation with ATP or Nig. Data are from three independent experiments with biological duplicates in each (mean ± SEM) or are representative of three independent experiments. Student’s *t*-test, **p < 0.01

Previous studies showed that ABHD5 deficiency and triglyceride accumulation stimulated ROS-dependent NLRP3 inflammasome activation in macrophages [24]. To further investigate the biological function of ABHD5 in NLRP3 inflammasome activation, specific and effective shRNA targeting ABHD5 was used to suppress the expression of endogenous ABHD5 in THP-1 macrophages (Fig. 4a). Caspase-1 cleavage is a critical step for the NLRP3 inflammasome activation. We then investigated the effects of ABHD5 on caspase-1 cleavage. After knockdown of ABHD5, NLRP3 inflammasome activation was strengthened, as more cleaved caspase-1 was detected in ABHD5-silenced macrophages treated by NLRP3 inflammasome activator such as ATP, Nigericin or Alum (Fig. 4a).

Similarly, interleukin (IL)-1β secretion was significantly increased in ABHD5 silenced macrophages primed by lipopolysaccharide (LPS) and then treated by NLRP3 inflammasome activator such as ATP, Nigericin or Alum (Fig. 4b). To confirm the inhibitory role of ABHD5 in IL-1β secretion, the effects of ABHD5 deficiency on IL-1β expression in mouse BMDMs were observed. ATP and Nigericin stimulated IL-1β secretion by LPS-primed ABHD5-deficient BMDMs was significantly increased (Fig. 4c). However, ABHD5 deficiency has no regulatory effects on IL-1β mRNA expression level (Fig. 4d). In contrast, ABHD5 overexpression greatly inhibited IL-1β secretion in THP-1 macrophages (Fig. 4e). We also detected the effect of ABHD5 on other inflammasome activation such as NLRC4 and AIM2 inflammasomes. However, the NLRC4 activator flagellin-induced or AIM2 activator poly (dA:dT)-induced IL-1β production was comparable between control and ABHD5-silenced macrophages (Fig. 4b, c). Results above showed that proteasomal degradation of ABHD5 is mediated by TRIM59. Thus, we overexpressed TRIM59 in THP-1 macrophages by infected with TRIM59 lentiviral particles. As expected, exogenous up-regulation of TRIM59 expression significantly promoted IL-1β release by following stimulation with ATP or Nigericin (Fig. 4f). Collectively, these data indicated that ABHD5 deficiency specifically promote NLRP3 inflammasome activation and subsequent IL-1β secretion.

### TRIM59 mediates the pro-tumorigenic effects of macrophages

TAMs are known to have an important role in tumor progression through the secretion of multiple pro-inflammatory cytokines and chemokines [13]. To determine whether macrophages educated by TRIM59 contribute to the promotion of tumor characteristics, we conducted a set of experiments in vitro.

**Fig. 5:**
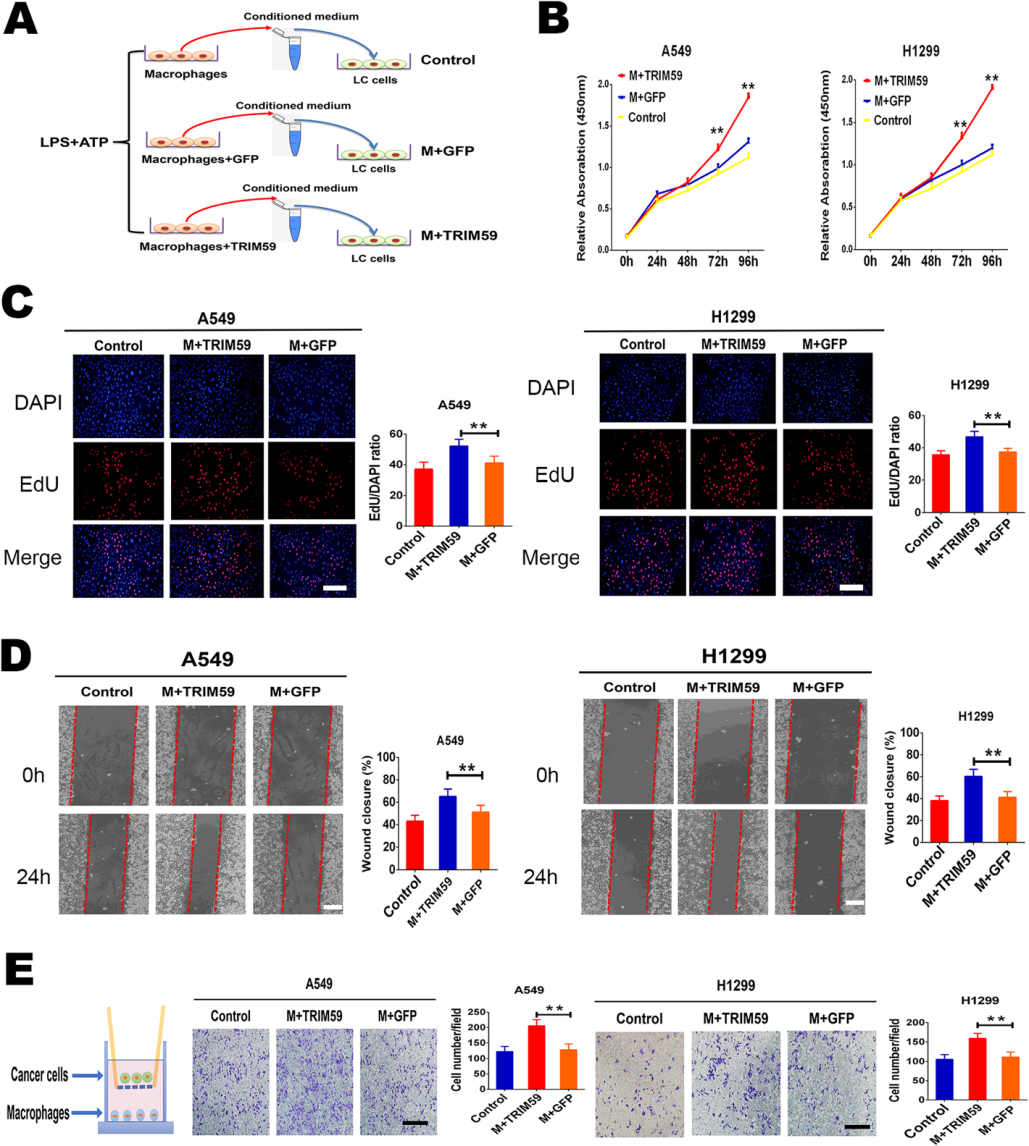
TRIM59 induces the macrophages to promote the proliferation, migration, and invasion of lung cancer cells. **a**. A schematic diagram illustrating the experimental design. THP-1 macrophages infected with lentiviral vectors expressing TRIM59 or GFP control primed with LPS for 8 h, and followed by stimulation with ATP. The effect of the conditioned medium of THP-1 macrophages on the proliferation and migration of lung cancer cells (H1299 and A549) was analyzed by CCK8, EdU analysis, and wound-healing assays. **b**. The effect of conditioned medium of macrophages infected with TRIM59 lentiviral particles on H1299 and A549 cells proliferation were measured by CCK8 assay. **c**. The effect of conditioned medium of macrophages infected with TRIM59 lentiviral particles on H1299 and A549 cells proliferation were measured by EdU analysis. Scale bars, 100 μm. **d**. Migration of lung cells educated by different the conditioned medium was assessed by wound healing assays. Representative results are shown, wound closure evaluated at 0 and 24 h after wounding. Quantification of wound healing percentage were performed with the Image J software. Scale bars, 200 μm. **e**. Invasion capacity of lung cancer cells cocultured with macrophages infected with TRIM59 lentiviral particles or GFP lentiviral control primed with LPS for 8 h, and followed by stimulation with ATP was determined by the in vitro transwell coculture system. Representative images were shown. Scale bars, 150 μm. All these experiments have been repeated three times. Data are presented as mean ± SEM. The significance was determined by Student’s *t*-test. ** *p* < 0.01

First, it was observed that TRIM59 can be delivered into the macrophages by exosomes derived from lung cancer cells. Furthermore, TRIM59 directly induced the ubiquitination of ABHD5 and led to its proteasome-dependent degradation, which activating NLRP3 inflammasome signaling pathway to promote IL-1β secretion in macrophages. Then, we used the vitro culture model to mimic the local tumor microenvironment, by using concentrated conditioned medium (CCM) from THP-1 macrophages infected with lentiviral vectors expressing TRIM59 or GFP control in the presence of the LPS and ATP stimulation (Fig. 5a). We observed that macrophages treated with TRIM59 resulted in a greater increase in A549 and H1299 cell proliferation compared to that observed following macrophage treated with control as indicated by CCK8 and the EdU assay (Fig. 5b, c). Consistent with these observations, the conditioned medium from macrophages infected with TRIM59 lentiviral particles showed potent stimulation of migration ability, whereas the conditioned medium from macrophages infected with GFP lentiviral control had a much weaker effect (Fig. 5d). In addition, invasion transwell assays showed that invasion abilities of H1299 and A549 cells were significantly increased by macrophages infected with TRIM59 lentiviral particles (Fig. 5e). These data provided strong evidence that macrophages educated by TRIM59 promote lung cancer progression.

### ABHD5 deficiency induces metabolic reprogramming in macrophages

**Fig. 6:**
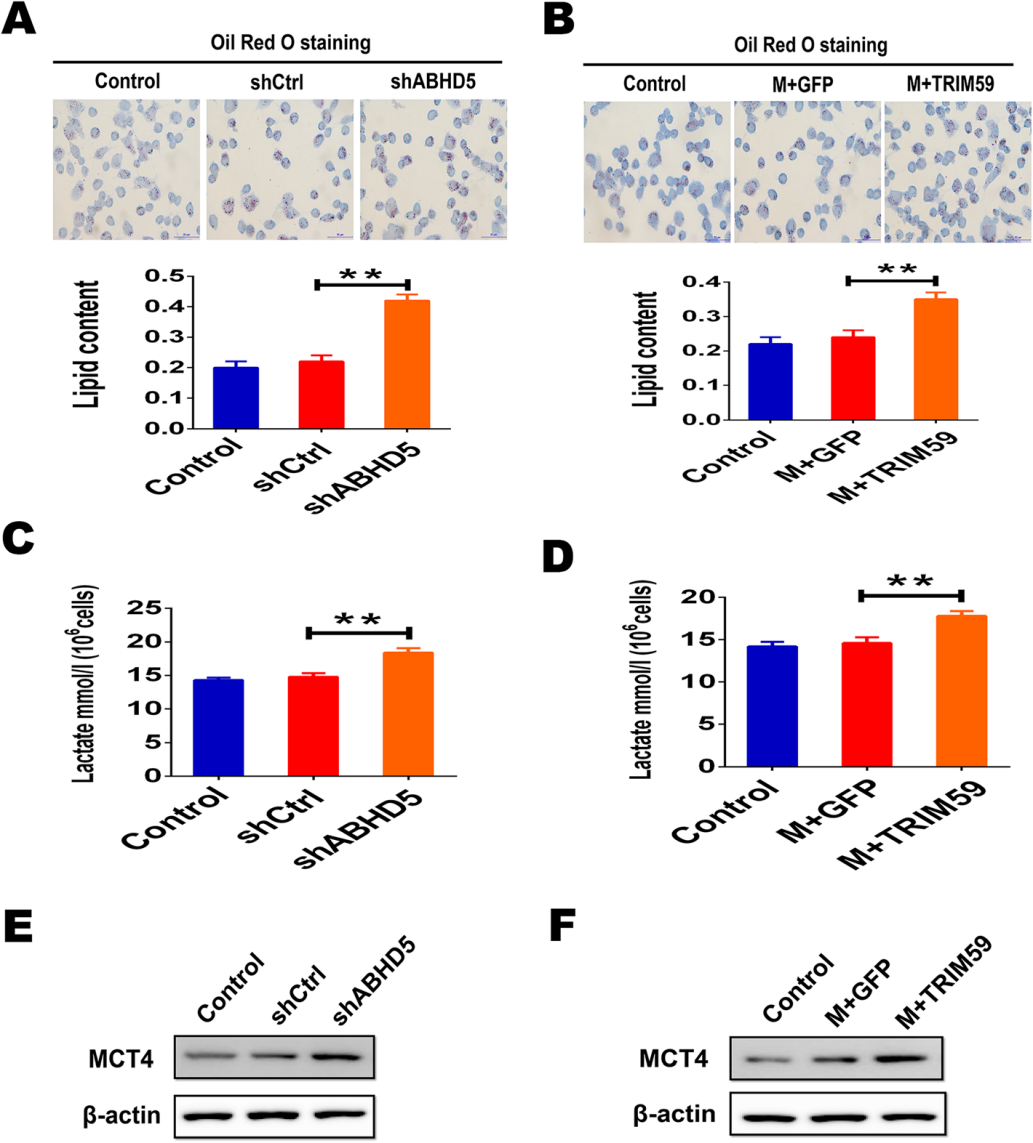
Loss of ABHD5 causes metabolic reprogramming in macrophages. **a**. THP-1 cells treated with shCtrl or shABHD5 and control were stained by oil red, representative photographs were taken, then analyzed lipid content by a quantitative method. Scale bars, 50 μm. **b**. THP-1 macrophages infected with lentiviral vectors expressing TRIM59 or GFP control and control stained by oil red, then analyzed lipid content by a quantitative method. Scale bars, 50 μm. **c**-**d**. THP-1 cells stably transfected with shCtrl or shABHD5 (**c**) or infected with TRIM59 lentiviral particles or GFP lentiviral control (**d**). Lactate secretion ability was measured. **e**-**f**. Protein level of MCT4 was analyzed by immunoblot. All statistic data in this figure represent mean ± SEM. The significance was determined by Student’s *t*-test. ** *p* < 0.01

The well-established function of ABHD5 is a co-activation of adipose triglyceride lipase (ATGL) in triglyceride hydrolysis [25]. Mutation or loss function of ABHD5 causes lipid accumulation in multiple organs or tissues [25]. From our previous findings, we speculated that TRIM59 promotes ABHD5 proteasomal degradation and impairs its lipolytic activity, thereby promoting macrophages metabolic reprogramming. Metabolic reprogramming in TAMs is associated with cancer development [26]. Thus, we sought to determine whether ABHD5 deficiency would promote macrophages metabolic reprogramming. We evaluated metabolic alteration of macrophages on the basis of two indicators: lipid droplets (LDs) content and lactate secretion. LDs are lipid-enriched cellular organelles. The accumulation of LDs is typically associated with pathogenic statuses such as oxidative stress, tissue injury and mitochondrial dysfunction [26, 27]. Oil Red O staining revealed that the level of accumulated LDs in THP-1 cells treated with ABHD5 shRNA significantly increased (Fig. 6a). Similarly, macrophages infected with TRIM59 lentiviral particles exhibited excessive LDs accumulation compared with macrophages infected with GFP lentiviral control (Fig. 6b). As shown in Fig. 6c, lactate secretion also significantly increased in ABHD5-deficient macrophages. In addition, THP-1 macrophages infected with TRIM59 lentiviral particles significantly promoted lactate secretion compared with macrophages infected with GFP lentiviral control (Fig. 6d). Consistently with these findings, the expression of the lactate transporter monocarboxylate transporter 4 (MCT4) expression was substantially increased in ABHD5-deficient or TRIM59 overexpressed THP-1 macrophages (Fig. 6e, f). These studies demonstrated that the metabolic profile of THP-1 macrophages shifted in ABHD5 deficiency macrophages.

### TRIM59 stimulates macrophages to facilitate lung cancer growth and metastasis in vivo

**Fig. 7:**
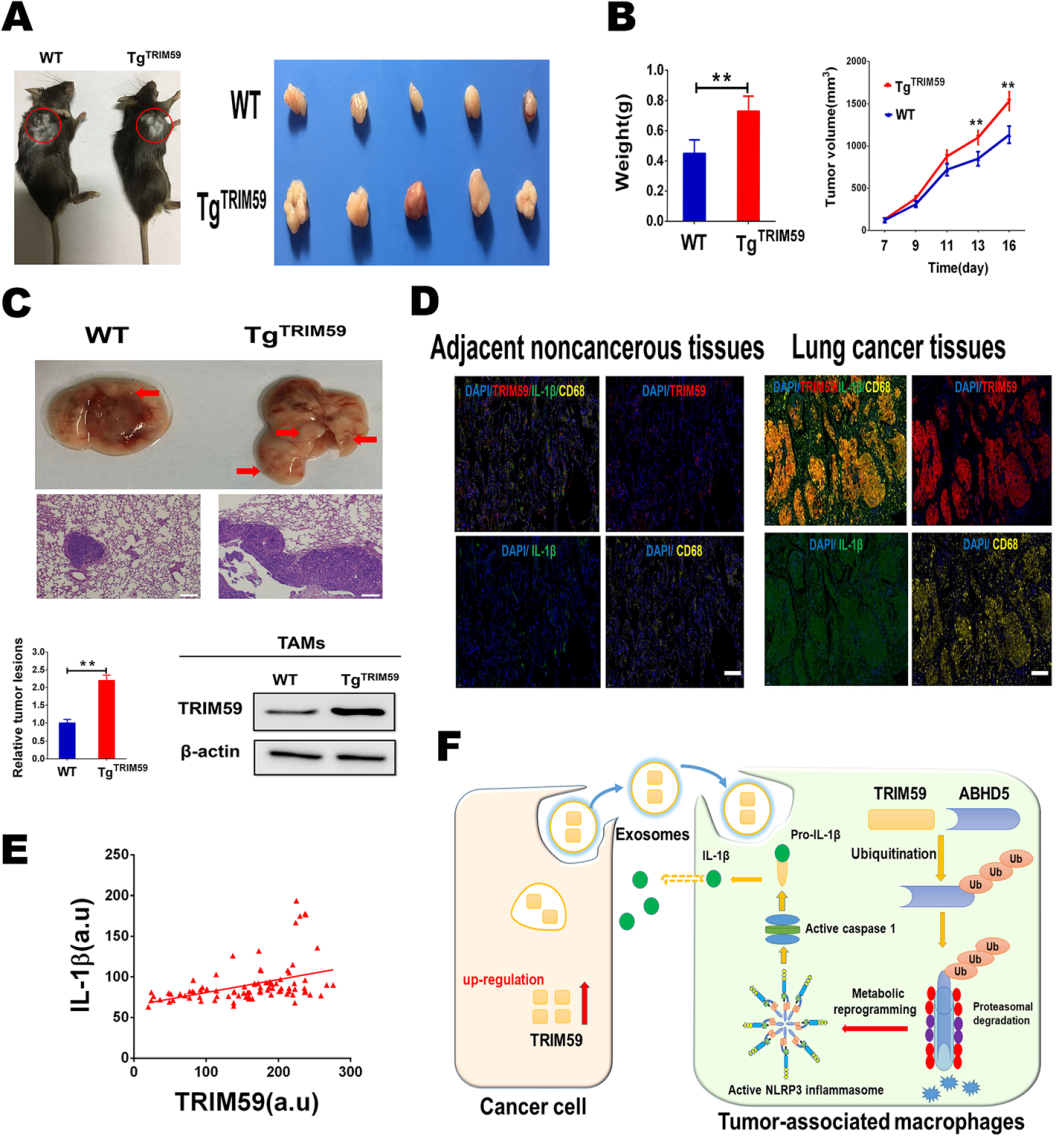
TRIM59 stimulates macrophages to facilitate tumor growth and metastasis in vivo. **a**. Representative image of tumor growth in WT and Tg^TRIM59^ mice subcutaneously inoculated with LLC cells (*n* = 5 per group). **b**. The growth curve of subcutaneous tumor in WT and Tg^TRIM59^ mice. And comparison of tumor weight from two groups at the end of the experiment. **c**. LLC cells (5.0 × 10^6^/100 μl of DMEM) were intravenously injected into WT and Tg^TRIM59^ mice via the tail vein (*n* = 6 in each group). Mice were sacrificed for further observation on day 14. Representative images of lung metastasis in WT and Tg^TRIM59^ mice. The total area of invasive lesions on the lung slice section represents the invasive tumor volume in the lungs. Immunoblotting assay of TRIM59 in TAMs from the lung metastases of WT or Tg^TRIM59^ mice. Scale bars, 200 μm. **d**. Fluorescent multiplex immunohistochemistry (mIHC) staining of adjacent noncancerous tissues and LC tissues. Representative image of an LC case was shown with TRIM59 and IL-1β co-expression (DAPI, blue; TRIM59, red; IL-1β, green; CD68, yellow). Scale bars, 200 μm. **e**. multiplex immunohistochemistry analysis of TRIM59 and IL-1β protein levels in lung cancer samples on tissue microarrays. The expression of TRIM59 and IL-1β expression in TAMs were measured with mean fluorescence intensities (MFIs) (in arbitrary units, a.u.), respectively. The Pearson correlation between TRIM59 and IL-1β expression (*n* = 90; *p* < 0.01, *r* = 0.414). **f**. Chematic illustration of the crosstalk between macrophages and cancer cells in the tumor microenvironment. Our data indicate that exosomal TRIM59 stimulates macrophages NLRP3 inflammasome activation to release IL1-β, which, in turn, promotes LC cells proliferation and invasion. All data are shown as mean ± SEM. Student’s t-test was used to analyze the data. **p < 0.01

To mimic the up-regulation of TRIM59 expression in TAMs and investigate its role in LC cells growth and metastasis in vivo, we utilized a CD11b promoter-driven macrophages-specific TRIM59 transgenic (Tg^TRIM59^) mouse model, in which the lewis lung carcinoma (LLC) cells were inoculated subcutaneously. Then, subcutaneous tumor growth was monitored and compared between the two groups. As shown in Fig. 7a and b, the tumor volumes of the Tg^TRIM59^ mice increased more quickly than those of the WT littermates. Accordingly, the net weight of the corresponding tumors was also significantly increased compared with control weights at termination of the experiment. Overexpression of TRIM59 was confirmed in the peritoneal macrophages (PMs) of the Tg^TRIM59^ mice compared with WT mice (Figure S2A). Moreover, no metastatic lesions was found in the lungs, liver and kidneys in two groups mice (Figure S2B).

To further assess the function of TRIM59 in macrophages, which stimulate the progression of lung cancer in vivo, LLC cells were injected into the C57BL/6 WT or transgenic mice via tail vein. After 2 weeks, we observed that Tg^TRIM59^ mice had more and larger nodules of metastatic lung tumors than WT mice (Fig. 7c). Consistent with this result, TAMs in the lung metastases in Tg^TRIM59^ mice had higher levels of TRIM59 mRNA and protein relative to that in the control group (Fig. 7c, Figure S2C). To further analyse the expression of TRIM59 and IL-1β at the protein level in TAMs, we performed the multiplex immunohistochemistry (mIHC) experiments in lung cancer tissue microarray, and found that TRIM59 expression in the TAMs was elevated as compared with macrophages in adjacent noncancerous tissues (Fig. 7d). Moreover, quantitative analysis indicated that TRIM59 expression positively correlated with IL-1β expression in TAMs (Fig. 7e). These results indicated that macrophages educated by TRIM59 facilitates lung cancer growth and metastasis in vivo. In sum, our results showed that, tumor-derived exosomal TRIM59 converts macrophages to tumor-promoting functions of macrophages via regulating ABHD5 proteasomal degradation, to activate NLRP3 inflammasome signaling pathway to promote lung cancer progression by IL-1β secretion (Fig. 7f). Our findings also indicated that tumor-derived exosomal TRIM59 has an important role in intercellular communication for fostering an inflammatory microenvironment and promoting lung metastasis.

## Discussion

Modification of the microenvironment allows tumor cells to overcome inhospitable extracellular conditions and progress towards metastasis. Recent studies have highlighted the key involvement of exosomes in the facilitation of tumor microenvironment [28, 29]. Exosomes convey information to neighboring or remote cells by delivering proteins and RNAs thus affecting signaling pathways in various physiological and pathological conditions including cancer [28]. Exosomes serve as communication vehicles between the tumor cells and their surroundings, favoring secretion of growth factors, cytokines, and angiogenic factors by stromal cells, fibroblasts, immune and inflammatory cells [30]. In this study, we demonstrated that interaction between LC cells and macrophages by exosomal TRIM59 results in dysregulated NLRP3 inflammasome activity that drive macrophages-mediated tumor progression. First, TRIM59 can be transferred by exosomes from tumor cells to macrophages. Second, exosomal transfer of TRIM59 directly interacts with ABHD5 and increases ubiquitin- and proteasome-mediated ABHD5 degradation. Finally, ABHD5 degradation triggers NLRP3 signaling activation in macrophages, which facilitates secretion of IL-1β, resulting in increased proliferation and migration potential of LC cells.

Emerging clinical evidence shows that TRIM59 were frequently up-regulated in tumor tissues compared with noncancerous tissues from breast and gastric tumors, where increased levels were correlated with advanced clinical stages and reduced survival among cancer patients [16, 31]. TRIM59 promotes tumor growth in hepatocellular carcinoma and regulates the cell cycle by degradation of protein phosphatase 1B [18]. In addition, TRIM59 as an essential tumor-promoting factor that facilitates breast cancer growth and metastasis through modulating PDCD10-associated signaling pathways [16]. It is now clear that cancer progression and metastasis depend on the bidirectional interactions between cancer cells and their environment, forming the tumor microenvironment. However, the pro-oncogenic role of TRIM59 in the tumor microenvironment have not yet been identified. In this study, exosomes secreted from lung cancer cells were isolated. We found that TRIM59 was detected in the exosomes of lung cancer cells, whereas the levels of TRIM59 in both lung cancer cells and exosomes were strongly decreased by transfection of shTRIM59. Moreover, these exosomes were found to enter into the macrophages in a dose and time-dependent manner. These results clearly demonstrated that the TRIM59 can be delivered into the macrophages by exosomes and implied that TRIM59 has functional roles in tumor microenvironment.

Macrophages represent a major component of the lympho-reticular infiltrates in solid tumors and play a crucial role in cancer progression [32]. Notably, it has been well accepted that TAMs in cancer tissues bear complicated characteristics rather than simply M2 or M1, and, it is noteworthy that TAMs exhibit non classical M2/M1 phenotypes [33]. In fact, a functional plasticity of TAMs has been proposed, wherein macrophages show an inflammatory phenotype in the early phase of tumor establishment, while displaying an immunosuppressive phenotype in the later phase of tumor progression [32]. Previous study suggested that TRIM59 loss in M2 macrophages promotes melanoma migration and invasion by upregulating MMP-9 and Madcam1 [34]. Interestingly, in this study, we demonstrated that macrophages were educated by TRIM59 promote lung cancer cells progression. When viewed in combination, it is tempting to speculate that the heterogeneity and dynamic nature of tumor microenvironment across different cancers, as well as different stages of the same cancer, it is quite likely that the function of TRIM59 in TAMs might also vary across these conditions. Thus, the precise regulatory mechanisms of TRIM59 expression in the macrophages require further exploration in future studies.

Recently, multiple lines of evidence suggest that ABHD5 acts as a tumor suppressor because it plays a role in inflammation and lipid metabolism in cancer [24]. Mechanistically, ABHD5 deficiency promotes colorectal tumor development by inducing glycolysis and epithelial-mesenchymal transition [24]. ABHD5 is an intracellular lipolytic activator whose deficiency causes lipid overload in most cells including macrophages [25, 35]. Metabolic adaptation is a key feature of macrophage plasticity and polarization and is instrumental to macrophage function in homeostasis, immunity, and inflammation [26, 27, 36]. In this study, we demonstrated that the metabolic profile of THP-1 macrophages shifted in ABHD5 deficiency macrophages. Emerging data support the concept that metabolic regulators control macrophages activation [26, 37]. Metabolic reprogramming in macrophages by certain stimulators is an important characteristic feature of TAMs that primarily affects their extrinsic regulatory functions in the tumor microenvironment [27]. Recent reports have suggested that ABHD5 was highly expressed in TAMs in the tumor microenvironment. Moreover, macrophage ABHD5 enhanced the growth of colorectal cancer cells by suppressing spermidine synthesis [38]. Interestingly, ABHD5 expression was heterogeneous in TAMs and that ABHD5^low^ macrophages (a macrophage subpopulation with decreased expression of ABHD5) potentially facilitate tumor cell migration and cancer metastasis [23]. However, ABHD5 expression downregulated in metastasis-associated TAMs are not fully understood. Herein, our results showed that the ubiquitination of ABHD5 by an E3 ubiquitin ligase, TRIM59, promoted ABHD5 proteasomal degradation in macrophages. Thus, we conclude that lung cancer cells secrete exosomal TRIM59 that directly targets ABHD5, leading to ABHD5 deficiency in TAMs. Our results extend our knowledge regarding the regulation of TRIM59 and potential targets for the development of novel therapeutic approaches targeting cancer metastasis.

It is well established that, an intimate relationship among IL-1β, the NLRP3 inflammasome and the metabolism of lipids and carbohydrates in TAMs [39]. IL-1β is a prominent proinflammatory cytokine, as it can efficiently cause the generation of other inflammatory mediators through signaling via IL-1 receptor, thus initiating a self-amplifying cytokine network [40]. IL-1β is considered to be indicators of an increased risk of carcinoma and poor prognoses in multiple cancers [41]. In the present study, we further revealed that the exacerbation of NLRP3 inflammasome activation by ABHD5 deficiency, provides a positive feedback loop to promote cancer progression by preferentially secrete the proinflammatory cytokine IL-1β. We have comprehensively linearized the TRIM59/ABHD5/NLRP3 signaling pathway and provided the concrete evidence showing such signaling events play essential roles in reprogramming macrophages and leading to the activation of oncogenic macrophages functions.

## Conclusion

Overall, our findings demonstrated an important role for tumor-derived exosomes promote the progression of lung cancer cells via the modulation of macrophages function. In addition, TRIM59/ABHD5/NLRP3 signaling axis plays an essential role in promoting pro-inflammatory cytokine IL-1β production and mediating metabolic reprogramming in macrophages. Understanding the bi-directional communication between tumor cells and macrophages as well as the regulation of tumor cells progression may lead to more effective strategies for macrophages-based cancer therapy.

## Supplementary information

**Additional file 1: Figure S1.** Exosomal TRIM59 is characteristically secreted by lung cancer cells and transferred to and internalized by macrophages via exosomes. **A-B.** Representative immunofluorescence image showed the internalization of PKH67-labeled A549-derived exosomes (green) by macrophages. Confocal imaging showed the delivery of PKH67-labeled exosomes (green) to macrophages. Green dots represented delivered exosomes. Scale bar, 150 μm. **C-D.** Green fluorescent protein (GFP)-tagged TRIM59 was expressed in H1299 and A549 cells and the LC cells-exosomes were isolated and incubated with THP-1 macrophages. The GFP-tagged TRIM59 was detected in THP-1 macrophages. Scale bar, 150 μm. **E-F.** THP-1 macrophages cells were incubated with exosomes from A549 for the noted periods of time or the noted doses. Western blot evaluations were used to evaluate TRIM59.

**Additional file 2: Figure S2.** Overexpression of TRIM59 in macrophages potentiates LC growth and metastasis in mice. **A.** TRIM59 mRNA levels increased in peritoneal macrophages (PMs) from Tg^TRIM59^ mice versus WT mice. LLC cells were subcutaneously injected to C57BL/6 WT and transgenic mice. Sixteen days later, PMs of tumor-free mice (NPM) or LLC tumor-bearing mice (TPM) were collected for mRNA assay with real-time PCR. Western blot evaluations were used to evaluate TRIM59. Histograms show means±s.e.m., **p < 0.01 (Student’s *t-*test). **B.** WT and Tg^TRIM59^ mice subcutaneously inoculated with LLC cells. All mice were euthanized at indicated time. Lung, liver, and kidney tissues of the indicated mice were harvested. Representative images of H&E stained lung, liver, and kidney sections are shown. In both groups of mice, no metastatic lesions was found in the lungs, liver and kidneys. Scale bar: 200 μm. **C.** TRIM59 mRNA levels increased in TAMs in the lung metastases from Tg^TRIM59^ mice versus WT mice. Histograms show means±s.e.m., **p < 0.01 (Student’s *t-*test).

## Data Availability

The datasets used and/or analyzed during the current study are available from the corresponding author on reasonable request.

## Abbreviations

LC: Lung cancer
TRIM59: Tripartite motif-containing 59
ABHD5: Abhydrolase domain containing 5
IL-1β: Interleukin-1β
NLRP3: NLR family protein containing a pyrin domain 3
TAMs: Tumor-associated macrophages
BMDMs: Bone marrow derived macrophages
LDs: Lipid droplets
LPS: Lipopolysaccharide
PLA: Proximity ligation assay
CHX: Cyclohexamide
