# Supplementary figures and images for: Cancer-derived exosomal TRIM59 regulates macrophage NLRP3 inflammasome activation to promote lung cancer progression
Source: J Exp Clin Cancer Res. 2020 Aug 31;39:176. doi: 10.1186/s13046-020-01688-7 (PMC7457778; doi:10.1186/s13046-020-01688-7)

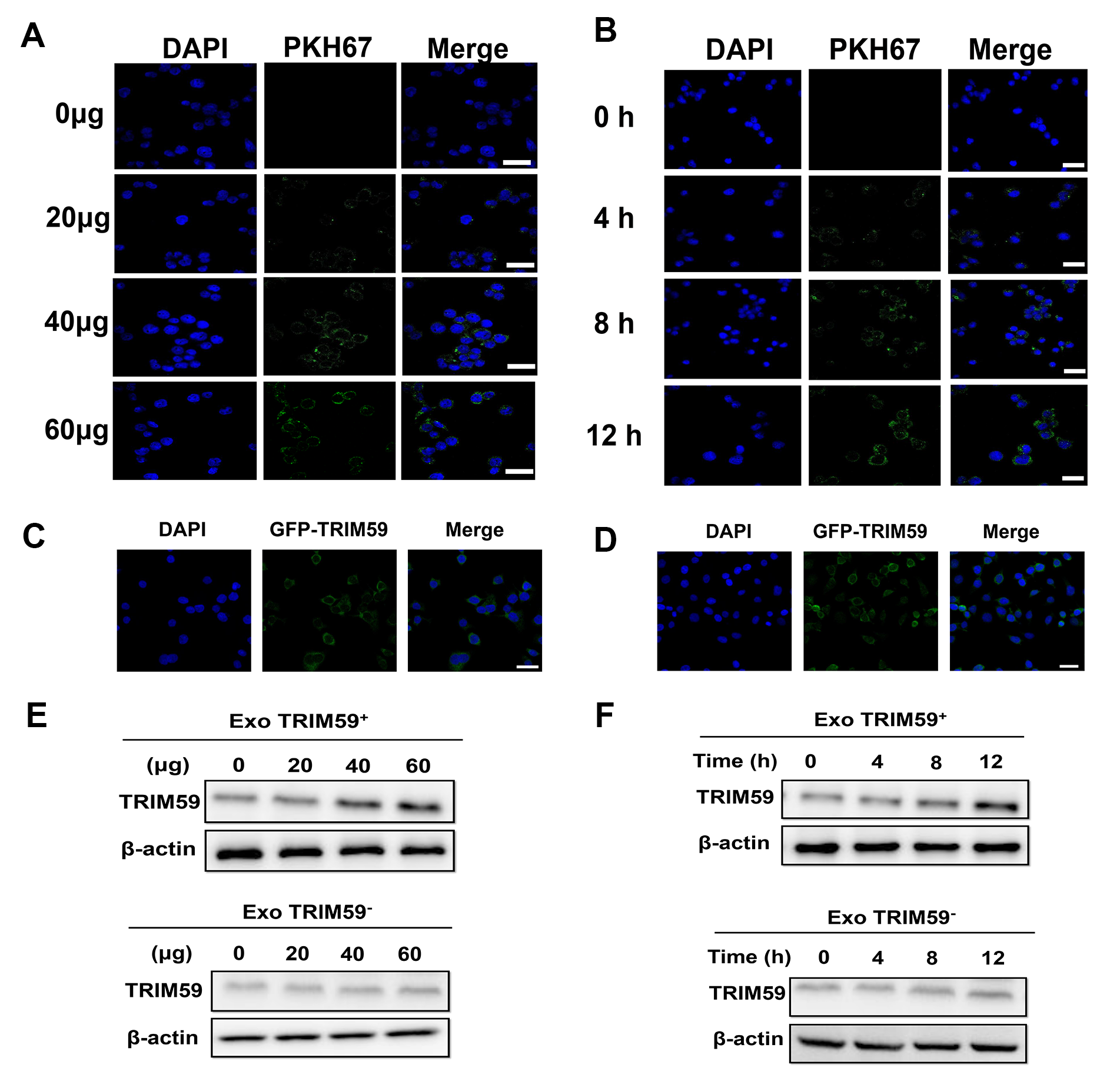

Supplement: Supplementary file 1 — Additional file 1: Figure S1. Exosomal TRIM59 is characteristically secreted by lung cancer cells and transferred to and internalized by macrophages via exosomes. A-B. Representative immunofluorescence image showed the internalization of PKH67-labeled A549-derived exosomes (green) by macrophages. Confocal imaging showed the delivery of PKH67-labeled exosomes (green) to macrophages. Green dots represented delivered exosomes. Scale bar, 150 μm. C-D. Green fluorescent protein (GFP)-tagged TRIM59 was expressed in H1299 and A549 cells and the LC cells-exosomes were isolated and incubated with THP-1 macrophages. The GFP-tagged TRIM59 was detected in THP-1 macrophages. Scale bar, 150 μm. E-F. THP-1 macrophages cells were incubated with exosomes from A549 for the noted periods of time or the noted doses. Western blot evaluations were used to evaluate TRIM59. [file 13046_2020_1688_MOESM1_ESM.tif]

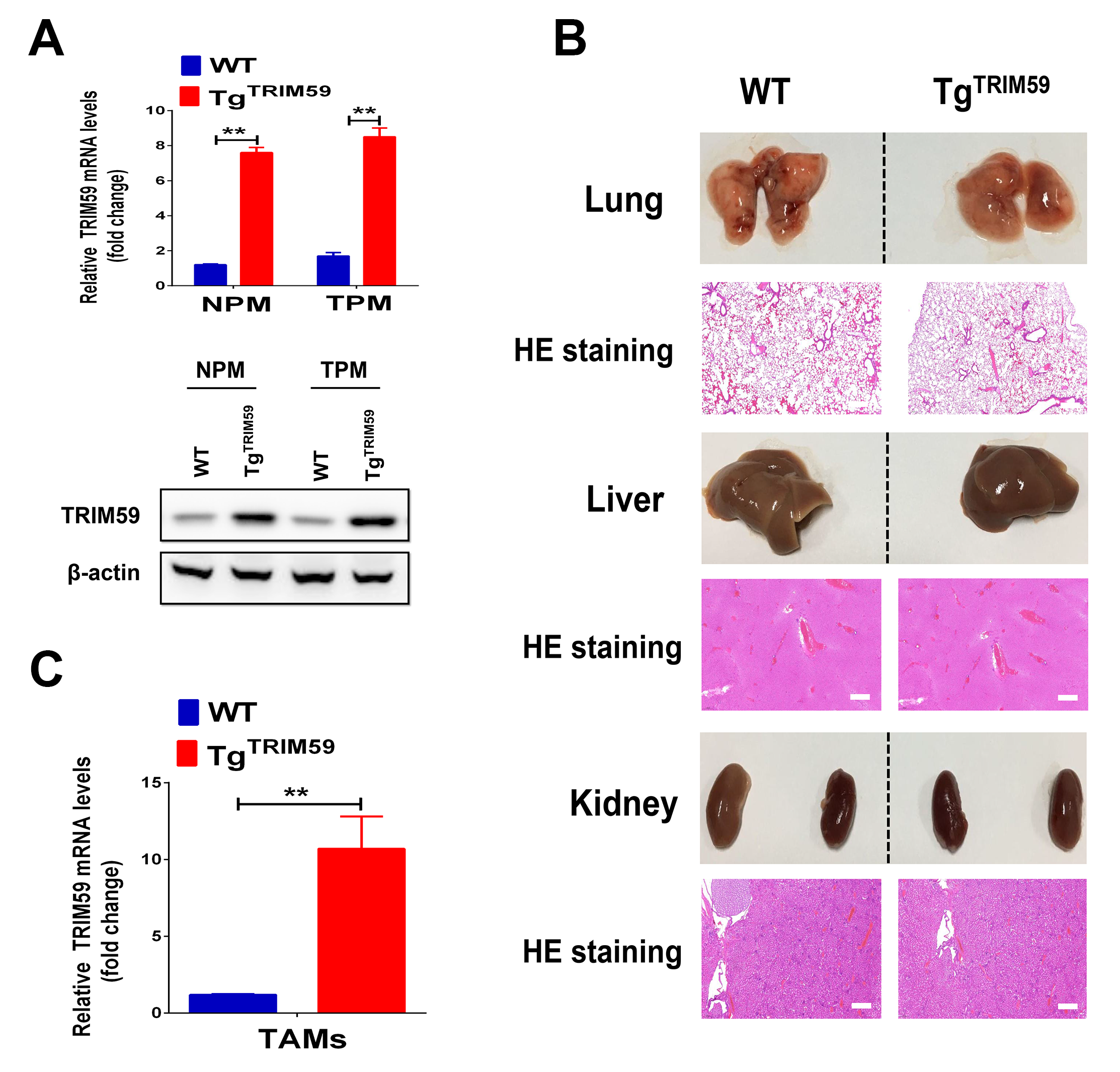

Supplement: Supplementary file 2 — Additional file 2: Figure S2. Overexpression of TRIM59 in macrophages potentiates LC growth and metastasis in mice. A. TRIM59 mRNA levels increased in peritoneal macrophages (PMs) from TgTRIM59 mice versus WT mice. LLC cells were subcutaneously injected to C57BL/6 WT and transgenic mice. Sixteen days later, PMs of tumor-free mice (NPM) or LLC tumor-bearing mice (TPM) were collected for mRNA assay with real-time PCR. Western blot evaluations were used to evaluate TRIM59. Histograms show means±s.e.m., **p < 0.01 (Student’s t-test). B. WT and TgTRIM59 mice subcutaneously inoculated with LLC cells. All mice were euthanized at indicated time. Lung, liver, and kidney tissues of the indicated mice were harvested. Representative images of H&E stained lung, liver, and kidney sections are shown. In both groups of mice, no metastatic lesions was found in the lungs, liver and kidneys. Scale bar: 200 μm. C. TRIM59 mRNA levels increased in TAMs in the lung metastases from TgTRIM59 mice versus WT mice. Histograms show means±s.e.m., **p < 0.01 (Student’s t-test). [file 13046_2020_1688_MOESM2_ESM.tif]
